# Predicting renal function recovery and short-term reversibility among acute kidney injury patients in the ICU: comparison of machine learning methods and conventional regression

**DOI:** 10.1080/0886022X.2022.2107542

**Published:** 2022-08-05

**Authors:** Xiujuan Zhao, Yunwei Lu, Shu Li, Fuzheng Guo, Haiyan Xue, Lilei Jiang, Zhenzhou Wang, Chong Zhang, Wenfei Xie, Fengxue Zhu

**Affiliations:** a Department of Intensive Care Medicine, Trauma Center, Peking University People’s Hospital, Beijing, PR China; b Department of Yunnan Baiyao Group Medicine Electronic Commerce Co., Ltd, Beijing, PR China

**Keywords:** Acute kidney injury, renal function recovery, renal function recovery time, machine learning

## Abstract

**Background:**

Acute kidney injury (AKI) is one of the most frequent complications of critical illness. We aimed to explore the predictors of renal function recovery and the short-term reversibility after AKI by comparing logistic regression with four machine learning models.

**Methods:**

We reviewed patients who were diagnosed with AKI in the MIMIC-IV database between 2008 and 2019. Recovery from AKI within 72 h of the initiating event was typically recognized as the short-term reversal of AKI. Conventional logistic regression and four different machine algorithms (XGBoost algorithm model, Bayesian networks [BNs], random forest [RF] model, and support vector machine [SVM] model) were used to develop and validate prediction models. The performance measures were compared through the area under the receiver operating characteristic curve (AU-ROC), calibration curves, and 10-fold cross-validation.

**Results:**

A total of 12,321 critically ill adult AKI patients were included in our analysis cohort. The renal function recovery rate after AKI was 67.9%. The maximum and minimum serum creatinine (SCr) within 24 h of AKI diagnosis, the minimum SCr within 24 and 12 h, and antibiotics usage duration were independently associated with renal function recovery after AKI. Among the 8364 recovered patients, the maximum SCr within 24 h of AKI diagnosis, the minimum Glasgow Coma Scale (GCS) score, the maximum blood urea nitrogen (BUN) within 24 h, vasopressin and vancomycin usage, and the maximum lactate within 24 h were the top six predictors for short-term reversibility of AKI. The RF model presented the best performance for predicting both renal functional recovery (AU-ROC [0.8295 ± 0.01]) and early recovery (AU-ROC [0.7683 ± 0.03]) compared with the conventional logistic regression model.

**Conclusions:**

The maximum SCr within 24 h of AKI diagnosis was a common independent predictor of renal function recovery and the short-term reversibility of AKI. The RF machine learning algorithms showed a superior ability to predict the prognosis of AKI patients in the ICU compared with the traditional regression models. These models may prove to be clinically helpful and can assist clinicians in providing timely interventions, potentially leading to improved prognoses.

## Introduction

Acute kidney injury (AKI) is one of the most common diseases with an incidence of 10–15% in inpatients [1]; in contrast, its morbidity can be as high as 50–60% in critically ill populations [2]. Despite advances in healthcare, the development of AKI is still independently associated with increased health care costs, the length of hospital stay, in-hospital morbidity, and mortality [3–5]. Unsurprisingly, the time for renal function recovery notably reflects the outcomes. A 2016 study of nearly 17,000 patients demonstrated that the persistence of AKI *versus* a prompt recovery is associated with higher morbidity and mortality [6]. Therefore, the severity of AKI and its timely treatment make AKI a consummate candidate for the use of predictive analytics.

Some scholars have pointed out that age, comorbidities, baseline renal function, and proteinuria have been shown to predict the probability of AKI recovery [7,8]. In addition, Srisawat et al. constructed a prediction model, which found that the APACHE-II score and Charlson comorbidity index were vital predictors [9]. However, their study only included a small group of patients (*n* = 76), which may reduce the accuracy of real-time implementation. Overall, the current research showed the present limitations in predicting whether the individual patient with AKI will recover and the recovery time.

Currently, to the best of our knowledge, there are only a few clinical studies that have included a significant number of patients and compared machine learning models to conventional regression models to predict renal function recovery and short-term reversibility after an episode of AKI. Therefore, it is hypothesized that our prognostic model will be accurate enough to recognize renal function recovery earlier among these vulnerable populations, so as to improve the prognosis of these patients by allowing for the increased opportunity to assist patients in the recovery from AKI and allowing for the prevention of further renal insults in the setting of an evolving injury, which may eventually lead to chronic kidney disease (CKD).

## Materials and methods

### Sources of data

This retrospective study was conducted by collecting data from an extensive critical care database named the Multiparameter Intelligent Monitoring in Intensive Care Database IV (MIMIC IV), which included all laboratory, medical test results, the pharmaceutical, and diagnostic codes for more than 40,000 ICU patients treated at Beth Israel Deaconess Medical Center (Boston, MA) from 2008 to 2019 [10]. To apply for access to the database, we completed the National Institutes of Health’s web-based course and successfully passed the Protecting Human Research Participants exam (No. 9936285). This study was approved by the institutional review board of Peking University People’s Hospital (Beijing Municipal Science, 7222199) and followed the Transparent Reporting of a Multivariable Prediction Model for Individual Prognosis and Diagnosis (TRIPOD) reporting guidelines.

### Selecting an AKI cohort

According to the Kidney Disease Improving Global Outcomes (KDIGO) clinical practice guidelines, we initially screened all adult patients who met the criteria for AKI within 48 h after ICU admission [11]. For exclusion criteria, patients who were discharged or died within 48 h after ICU admission and those who stayed in the ICU for more than 90 d were excluded. In addition, we excluded the cohort of participants who died with renal function recovery. The additional exclusion criteria included a history of receiving long-term renal replacement treatment (RRT), a diagnosis of advanced CKD, no data regarding the creatinine values after the AKI diagnosis, and when the patient’s maximum serum creatinine (SCr) was smaller than the baseline SCr.

### Data collection and definition

Data were abstracted from MIMIC IV using the Structured Query Language (SQL) with Navicat Premium (version 12.0.28). We obtained the demographic and clinical data within the first 24 h after ICU admission and the diagnosis of AKI. The comorbidities and diagnoses were identified based on the ICD-9 codes. The scoring systems included the Glasgow Coma Scale (GCS) score, Simplified Acute Physiology Score II (SAPS II), and Sequential Organ Failure Assessment (SOFA) score. The patient’s vital signs, including systolic blood pressure (SBP), heart rate (HR), peripheral oxygen saturation (SPO_2_), and temperature (T), were extracted. Furthermore, the patient’s laboratory data, including hemoglobin, leucocytes, basophils, monocytes, platelet count, lactate, albumin, anion gap, blood urea nitrogen (BUN), chloride, base excess, prothrombin time, and bicarbonate, were also recorded. In addition, the variables associated with AKI diagnosis were also abstracted, such as the urine volume and urine volume to weight ratio. Data regarding any therapy, such as vasopressors, antibiotics, furosemide, nephrotoxic drugs, mechanical ventilation, and RRT were also collected. Because of the high sampling frequency, we used the minimum, maximum, and mean values when extracting the vital signs and laboratory data.

In this study, we recognized that patients with renal function recovery no longer fulfilled the criteria for stage 1 AKI, but that their SCr levels might not have yet returned to baseline (defined as the return to <30% above the baseline) before being discharged from the ICU [12,13]. An alternative definition of non-recovery was the presence of meeting the AKI criteria or dying during the ICU stay. In addition, the AKI start time was the first time that the patient met the KDIGO criteria. The AKI recovery time was measured as the AKI recovery time minus the start time. We defined renal function recovery from AKI within 72 h of the initiating event typically recognized as the short-term reversal of AKI.

### Statistical analysis

The patients were divided into two groups based on whether they achieved renal function recovery, and the variables were displayed and compared between the groups. The demographics and other characteristics were summarized using means and standard deviations, medians, interquartile ranges, or frequency counts and percentages. The chi-squared test was used to compare the categorical variables, and the Mann–Whitney U test was used to compare the discrete distributions. The continuous variables were tested by the independent t-test. All of the data were analyzed using Python version 3.8 and R 4.0.5 (The R Foundation for Statistical Computing, Vienna, Austria) statistical software, with the statistical significance set at a *p* value <.05.

### Development and validation prediction model

We randomly separated the model development data into two parts: we used 90% of the data for the model derivation and 10% of the data for the internal validation. We developed a conventional logistic regression, XGBoost algorithm model, Bayesian networks (BNs), random forest model (RF), and support vector machine model (SVM) based on the training dataset, and verified these models in the validation dataset to identify the optimal predictors.

In the conventional method, each risk factor was used in the univariate analysis, and then a multivariate analysis was conducted to build the best fit logistic regression model. XGBoost is based on the sparsity-aware algorithm and is a weighted quantile sketch, in which the weak learners can be converged sequentially into the ensemble to achieve a strong learner [14]. The BN is a graphical representation, where each node corresponds to the random variables, and each edge represents the corresponding random variables' conditional probability [15]. RF is a learning method that unifies the results of multiple decision trees that are constructed based on the bootstrap sampling of the training dataset and randomly selects properties in each tree as a subset of the entire set of predictors [16]. SVM is an optimal classification algorithm in high-dimensional space to distinguish between different categories of samples, with the ability to transform training data into a high-dimensional feature space and make a linear optimal solution by separating a hyperplane that engages the smallest distance between the hyperplane points and the largest margin between the classes [17].

### Evaluating the performance of the models

To assess the model quality, we chose the area under the receiver operating characteristic curve (AU-ROC) as the measurement to compare the performances of the logistic regression and the machine learning algorithm models. In addition, we employed 10-fold cross-validation, which provides a more stable and reliable way to measure the performances of models. To further assess the models’ performances, a plot of the percentage of observations above a probability threshold *versus* the percentage of observations was constructed; then, an evaluation of the secondary metrics of the clinical prediction models, including the accuracy, sensitivity, specificity, precision, and recall, was performed [18].

## Results

### The demographics, clinical characteristics, and AKI metric measurements

**Figure 1.**
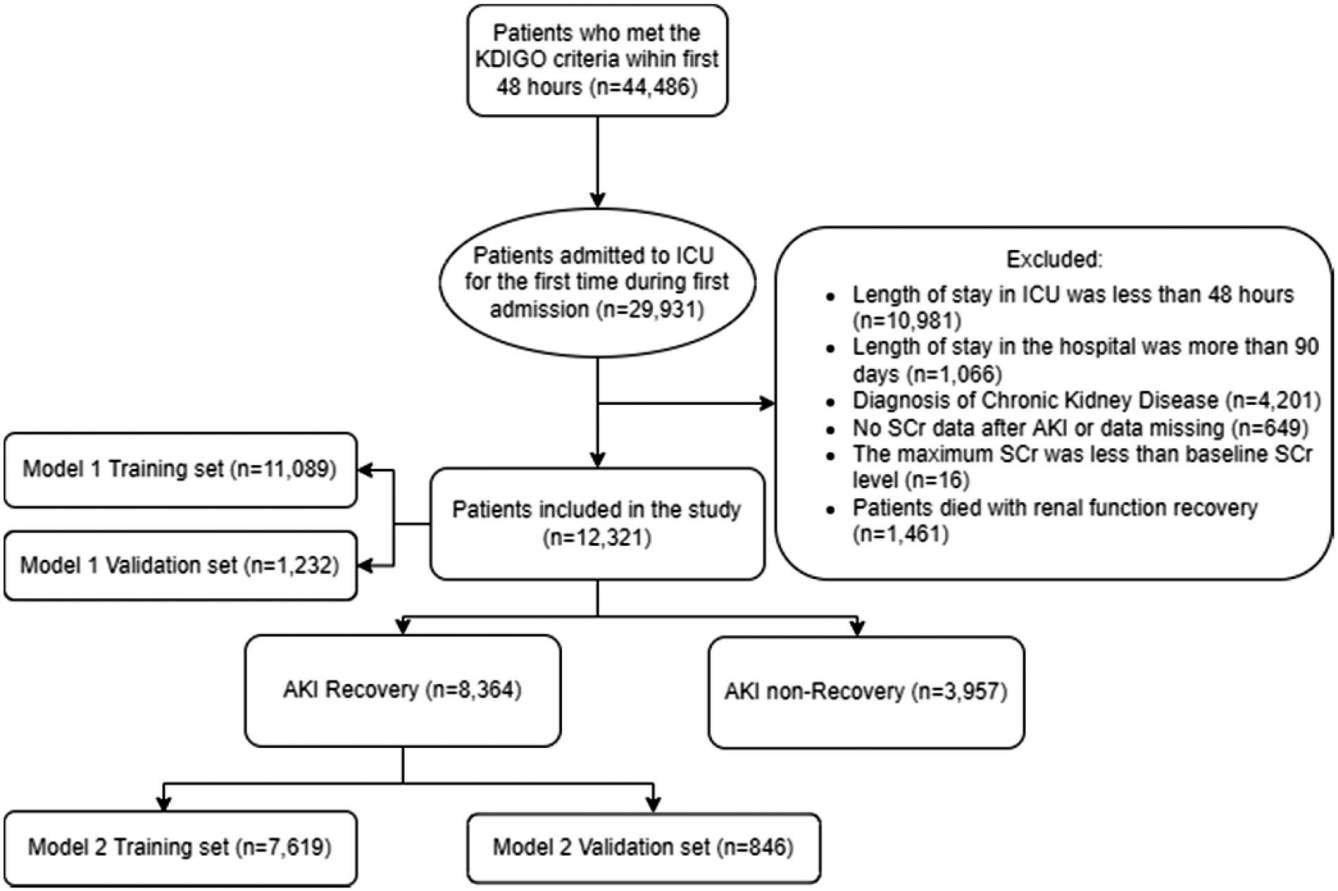
Flowchart depicting the number of critically ill patients included in the analysis after applying the exclusion criteria.

In total, 44,486 patients met the KDIGO criteria within the first 48 h after ICU admission. After excluding the patients according to the exclusion criteria, the final analysis cohort consisted of 29,931 eligible patients (Figure 1). In the analytic cohort, the average age of the patients was 66.7 years old; male patients accounted for 56.0% (*n* = 6901) of the cohort; white patients accounted for 67.9% (*n* = 8366) of the cohort, and the minimum GCS score was 13. Of these, the cohort was divided into two groups: the AKI recovery group (*n* = 8364, 67.9%) and the AKI non-recovery group (*n* = 3957, 32.1%). Of the recruited patients, 9460 patients were in stage 1 (6534 AKI recovery *versus* 2926 AKI non-recovery), 2634 were in stage 2 (1716 AKI recovery *versus* 918 AKI non-recovery), and 227 were in stage 3 (114 AKI recovery *versus* 113 AKI non-recovery). The baseline demographics, clinical characteristics, interventions, and outcomes are outlined in Table 1.

**Table 1.** Distribution of the baseline characteristics between the AKI recovery and non-recovery groups.

Characteristic	Total (*n* = 12,321)	AKI recovery (*n* = 8364)	AKI non-recovery (*n* = 3957)	*p*
Age (years), M (P_25_–P_75_)	66.7 (55.3–77.5)	66.3 (55.0–76.9)	67.6 (56.1–79.3)	.492
Male gender, *n* (%)	6901 (56.0)	4615 (55.2)	2286 (57.7)	.007
Ethnicity, *n* (%)				.039
White	8366 (67.9)	5714 (68.3)	2652 (67.0)	–
Black	873 (7.1)	569 (6.8)	304 (7.7)	–
Hispanic	365 (2.9)	253 (3.0)	112 (2.8)	–
Asian	269 (2.2)	173 (2.1)	96 (2.4)	–
Other	2225 (18.1)	1504 (17.9)	721 (18.2)	–
BMI (kg/m^2^)	27.2 (26.8–27.7)	27.2 (26.0–27.2)	27.2 (27.1–27.2)	.989
Comorbidities, *n* (%)				
Cardiovascular disease	9576 (77.7)	6546 (78.3)	3030 (76.6)	<.001
Chronic pulmonary disease	3097 (25.1)	2155 (25.7)	942 (23.8)	.020
Diabetes	3192 (25.9)	2104 (25.2)	1088 (25.2)	.006
Sepsis, *n* (%)	8122 (65.9)	5425 (64.9)	2697 (68.1)	<.001
Scoring systems, M (P_25_–P_75_)				
GCS min	13 (9–14)	14 (9–15)	13 (9–14)	<.001
SAPS II	36 (28–45)	41 (31–51)	34 (27–42)	<.001
SOFA > 4	8910 (72.3)	5813 (69.5)	3097 (78.3)	<.001
Vital signs, *n* (%)				
HR mean 24 h (bpm)	85.0 (75.5–96.9)	84.4 (74.9–96.1)	86.1 (76.4–98.9)	.410
SBP min<90 (mmHg)	6636 (53.9)	4357 (52.1)	2279 (57.6)	<.001
MAP min 24 h (mmHg)	58 (52–65)	59 (52–65)	57 (50–64)	<.001
T mean 24 h (°C)	36.9 (36.6–37.2)	36.9 (36.7–37.2)	36.8 (36.6–37.1)	.056
SPO_2_ first mean	97.3 (95.9 − 98.6)	97.4 (96.0–98.7)	97.1 (95.7–98.5)	.010
Urinary variables, M (P_25_–P_75_)				
Urine volume 24 h	1485 (965–2200)	1570 (1090–2300)	1240 (690–1980)	<.001
Urine volume to weight ratio 24 h	0.7 (0.5–1.0)	0.6 (0.4–0.9)	0.7 (0.5–1.0)	.090
Post-AKI urine volume max 24 h	3012 (1990–4395)	3300 (2320–4650)	2340 (1350–3625)	<.001
Post-AKI urine volume min 24 h	180 (75–500)	200 (90–550)	130 (45–400)	<.001
Serum laboratory variables, M (P_25_–P_75_)				
Baseline SCr (mg/dL)	0.7 (0.5–0.8)	0.6 (0.5–0.8)	0.8 (0.6–1.0)	<.001
SCr min 12 h (mg/dL)	0.9 (0.7–1.2)	0.9 (0.7–1.0)	1.1 (0.8–1.7)	<.001
SCr min 24 h (mg/dL)	0.8 (0.7–1.1)	0.8 (0.6–1.0)	1.1 (0.8–1.6)	<.001
Post-AKI SCr min 24 h (mg/dL)	0.7 (0.6–1)	0.7 (0.5–0.8)	1.0 (0.7–1.5)	<.001
Post-AKI SCr max 24 h (mg/dL)	1.1 (0.8–1.5)	1.0 (0.8–1.2)	1.7 (1.0–3.0)	<.001
Hemoglobin min 24 h (g/dL)	10.1 (8.5–11.8)	10.3 (8.7–11.9)	9.6 (8.1–11.4)	<.001
Leucocyte max 24 h (× 10^9^/L)	13.8 (10.1–18.7)	13.7 (10.2–18.3)	14.1 (10.0–19.5)	<.001
Platelet min 24 h (× 10^9^/L)	167.0 (117.0–229.0)	172.0 (124.0–233.3)	156.0 (102.0–220.0)	<.001
Basophils min 24 h (× 10^9^/L)	0.1 (0.0–0.3)	0.1 (0.0–0.5)	0.1 (0.0–0.2)	.001
Monocyte max 24 h (× 10^9^/L)	15.2 (1.3–31.7)	15.2 (1.3–30.0)	15.2 (1.4 − 37.0)	.036
Albumi*n* < 3.5 (g/dL)	3341 (27.1)	1950 (23.3)	1391 (35.2)	<.001
BUN max 24 h (mg/dL)	19 (14–29)	18 (14–24)	26 (17–41)	<.001
Lactate max 24 h (mmol/L)	2.3 (2.0–2.7)	2.3 (1.9–2.6)	2.3 (2.2–3.0)	<.001
Anion Gap min 24 h (mmol/L)	12 (1014)	12 (10–14)	13 (11–16)	<.001
Base excess min 24 h (mmol/L)	−3 (-4--1)	−3 (-4-0)	−3 (-6--2)	<.001
Prothrombin time min 24 h (sec)	28.5 (25.8–32.4)	28.5 (25.5–31.5)	29.3 (26.4–34.4)	<.001
Sodium min 24 h (mmol/L)	137 (135–140)	137 (135–140)	137 (134–139)	<.001
Chloride min 24 h (mmol/L)	103 (99–106)	103 (100–106)	102 (98–106)	<.001
Bicarbonate max 24 h (mmol/L)	24 (22–27)	24 (22–27)	23 (21–26)	<.001
Medications, *n* (%)				
Vancomycin	7578 (61.5)	4991 (59.7)	2587 (65.4)	<.001
Antibiotic duration	3 (2–4)	3 (2–5)	3 (1–4)	<.001
Nephrotoxic drug	10158 (82.4)	6852 (81.2)	3306 (83.5)	.029
Vasopressin	1191 (9.7)	555 (6.6)	636 (16.1)	<.001
Phenylephrine	4017 (32.6)	2753 (32.9)	1264 (31.9)	.292
Epinephrine	927 (7.5)	573 (6.9)	354 (8.9)	<.001
Furosemide	8373 (67.9)	5756 (68.8)	2617 (66.1)	.003
Intervention, *n* (%)				
Post-AKI ventilation 24 h	7735 (62.8)	5346 (63.9)	2389 (60.4)	<.001
RRT 24 h	264 (2.1)	28 (0.3)	236 (5.9)	<.001
Post-AKI RRT 24 h	253 (2.1)	25 (0.3)	228 (5.8)	<.001
AKI stage, *n* (%)				<.001
1	9460 (76.8)	6534 (78.1)	2926 (73.9)	–
2	2634 (21.4)	1716 (20.5)	918 (23.2)	–
3	227 (1.8)	114 (1.4)	113 (2.9)	–

AKI: acute kidney disease; BUN: blood urea nitrogen; HR: heart rate; MAP: mean arterial pressure; RRT: renal replacement therapy; SAPS II: Simplified Acute Physiology Score II; SBP: systolic pressure; SCr: serum creatinine; SOFA Score: Sepsis-Related Organ Failure Assessment Score; SPO_2_: peripheral oxygen saturation; T: temperature.

### The features selected and model comparison in renal function recovery

The results of the logistic regression analysis were outlined in Table 2. As expected, patients who had higher SAPS II (OR = 1.00, 95% CI 0.99–1.00), lower mean HR within 24 h (OR = 0.99, 95% CI 0.99–1.00), higher mean temperature within 24 h (OR = 0.12, 95% CI 0.10–0.15), lower SCr within 24 h of AKI diagnosis (OR = 0.54, 95% CI 0.50–0.59), higher hemoglobin within 24 h (OR = 1.07, 95% CI 1.04–1.09), lower anion gap within 24 h (OR = 0.97, 95% CI 0.95–0.98), shorter prothrombin time within 24 h (OR = 0.99, 95% CI 0.99–1.00), higher sodium level within 24 h (OR = 1.02, 95% CI 1.01–1.03), prolonged antibiotic duration (OR = 1.07, 95% CI 1.05–1.09), and lower AKI stage (OR = 0.72, 95% CI 0.65–0.80) demonstrated increased odds of failing to recover from AKI.

**Table 2.** The univariate and multivariate logistic regression analyses for renal function recovery.

	Univariate analysis		Multivariate analysis	
Variables	OR (95% CI)	*p*	OR (95% CI)	*p*
Age	0.99 (0.99–1.00)	<.001	–	–
Male gender	0.92 (0.84–0.99)	.034	–	–
Ethnicity	0.99 (0.97–1.02)	.607	–	–
BMI	1.01 (0.99–1.02)	.304	–	–
Cardiovascular disease	0.88 (0.84–0.91)	<.001	–	–
Chronic pulmonary disease	1.11 (1.00–1.21)	.033	–	–
Diabetes	0.87 (0.79–0.95)	.002	–	–
Sepsis	0.87 (0.79–0.95)	.001	–	–
GCS min	1.02 (1.00–1.03)	.006	–	–
SAPS II	0.96 (0.96–0.97)	<.001	1.00 (0.99–1.00)	.028
SOFA > 4	0.63 (0.57–0.69)	<.001		
HR mean 24 h	0.99 (0.99–1.00)	<.001	0.99 (0.99–1.00)	<.001
SBP min <90	1.01 (1.00–1.01)	<.001		
MAP min 24 h	1.01 (1.00–1.01)	<.001		
T mean 24 h	1.50 (1.39–1.62)	<.001	1.25 (1.14–1.37)	<.001
SPO_2_ first mean	1.07 (1.05–1.09)	<.001		
Urine volume 24 h	1.00 (1.00–1.00)	<.001		
Urine volume to weight ratio 24 h	1.01 (1.00–1.02)	<.001	0.12 (0.10–0.15)	<.001
Post-AKI urine volume min 24 h	1.00 (1.00–1.00)	<.001	–	–
Baseline SCr	0.07 (0.06–0.08)	<.001	–	–
SCr min 12 h	0.24 (0.21–0.26)	<.001	–	–
SCr min 24 h	0.18 (0.16–0.20)	<.001	–	–
Post-AKI SCr min 24 h	0.04 (0.03–0.05)	<.001	–	–
Post-AKI SCr max 24 h	0.28 (0.26–0.30)	<.001	0.54 (0.50–0.59)	<.001
Hemoglobin min 24 h	1.11 (1.09–1.13)	<.001	1.07 (1.04–1.09)	<.001
Leucocyte max 24 h	0.99 (0.98–0.99)	<.001	–	–
Platelet min 24 h	1.00 (1.00–1.00)	<.001	–	–
Basophils min 24 h	1.00 (0.98–1.01)	.655	–	–
Monocyte max 24 h	1.00 (0.99–0.99)	<.001	–	–
BUN max 24 h	0.96 (0.95–0.96)	<.001	–	–
Lactate max 24 h	0.88 (0.87–0.90)	<.001	–	–
Anion Gap min 24 h	0.87 (0.86–0.88)	<.001	0.97 (0.95–0.98)	<.001
Base excess min 24 h (mmol/L)	1.08 (1.07–1.09)	<.001		
Prothrombin time min 24 h	0.98 (0.97–0.98)	<.001	0.99 (0.99–1.00)	.001
Sodium min 24 h	1.03 (1.02–1.03)	<.001	1.02 (1.01–1.03)	.001
Chloride min 24 h	1.02 (1.01–1.03)	<.001	–	–
Bicarbonate max 24 h	1.09 (1.08–1.10)	<.001	–	–
Vancomycin	0.78 (0.72–0.85)	<.001	–	–
Antibiotic duration	1.03 (1.02–1.04)	<.001	1.07 (1.05–1.09)	<.001
Nephrotoxic drug	0.89 (0.79–0.98)	.025	–	–
Post-AKI ventilation 24 h	1.17 (1.07–1.27)	<.001	–	–
AKI Stage	0.78 (0.72–0.84)	<.001	0.72 (0.65–0.80)	<.001

AKI: acute kidney disease; BUN: blood urea nitrogen; HR: heart rate; MAP: mean arterial pressure; RRT: renal replacement therapy; SAPS II: Simplified Acute Physiology Score II; SBP: systolic pressure; SCr: serum creatinine; SOFA Score: Sepsis-Related Organ Failure Assessment Score; SPO_2_^:^ peripheral oxygen saturation; T: temperature.

**Figure 2.**
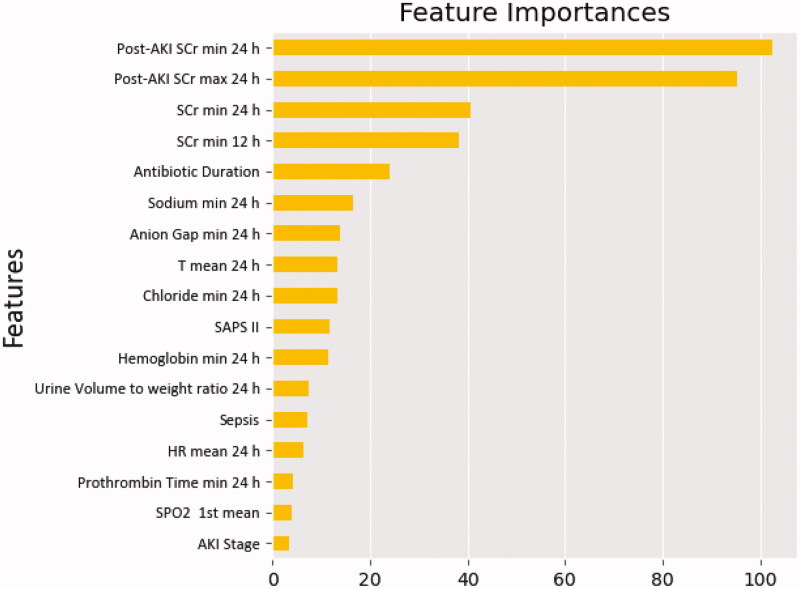
Importance of the matrix plot of the AKI predictors in the random forest model among critically ill patients. AKI: acute kidney disease; HR: heart rate; SAPS II: Simplified Acute Physiology Score II; SCr: serum creatinine; SpO2: peripheral oxygen saturation; T: temperature.

By comparing the performance of four different machine learning models, RF presented the best prediction value. According to the analysis results of each feature’s contribution by the RF model, the maximum and minimum SCr within 24 h from the diagnosis of AKI, the minimum Scr within 24 h, the minimum Scr within 12 h, and antibiotic duration were the top five essential predictors for predicting renal function recovery (Figure 2).

**Figure 3.**
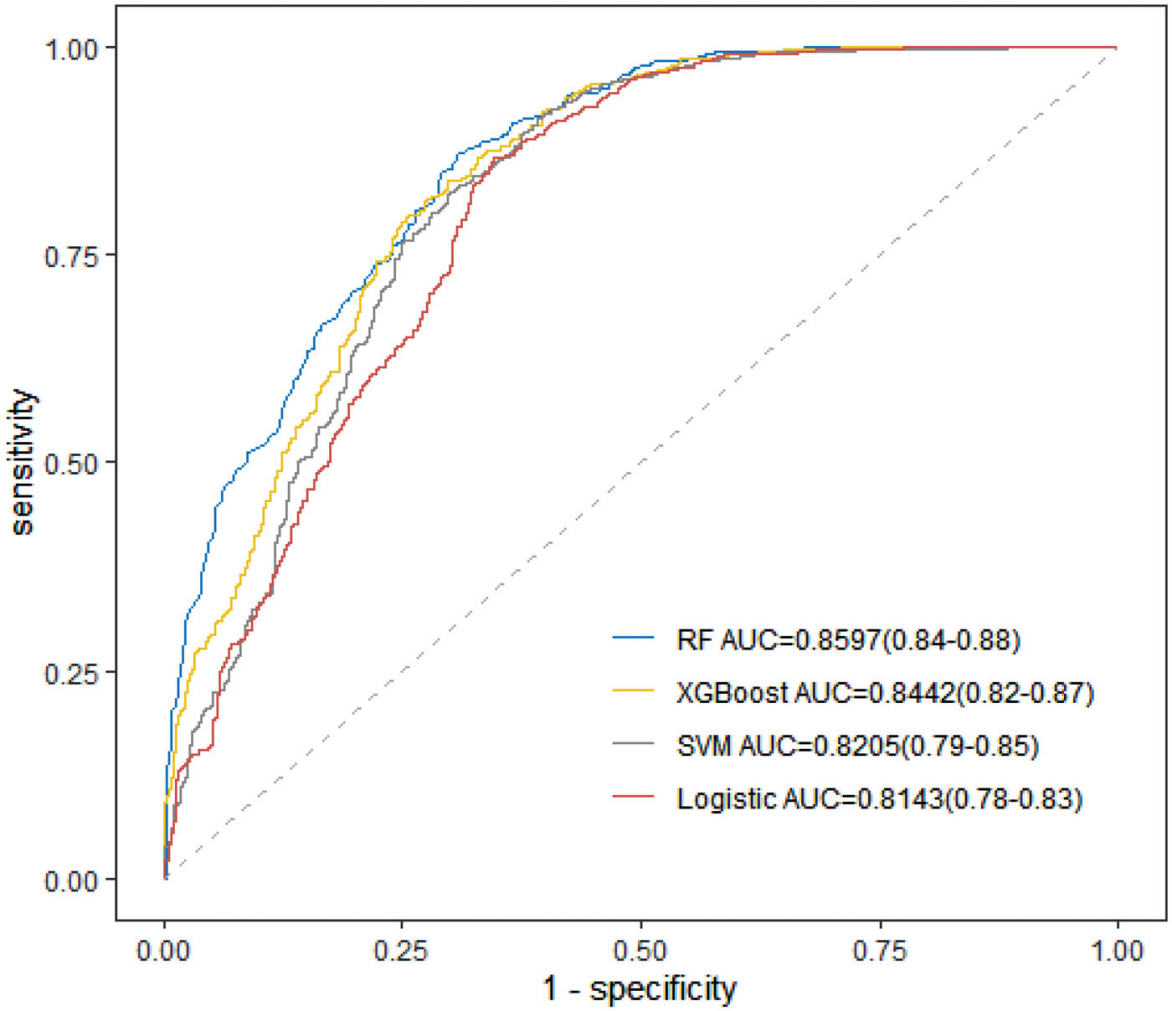
Receiver operating characteristic curve for estimating the discrimination of the logistic regression model, XGBoost model, random forest model (RF), and support vector machine model (SVM).

**Figure 4.**
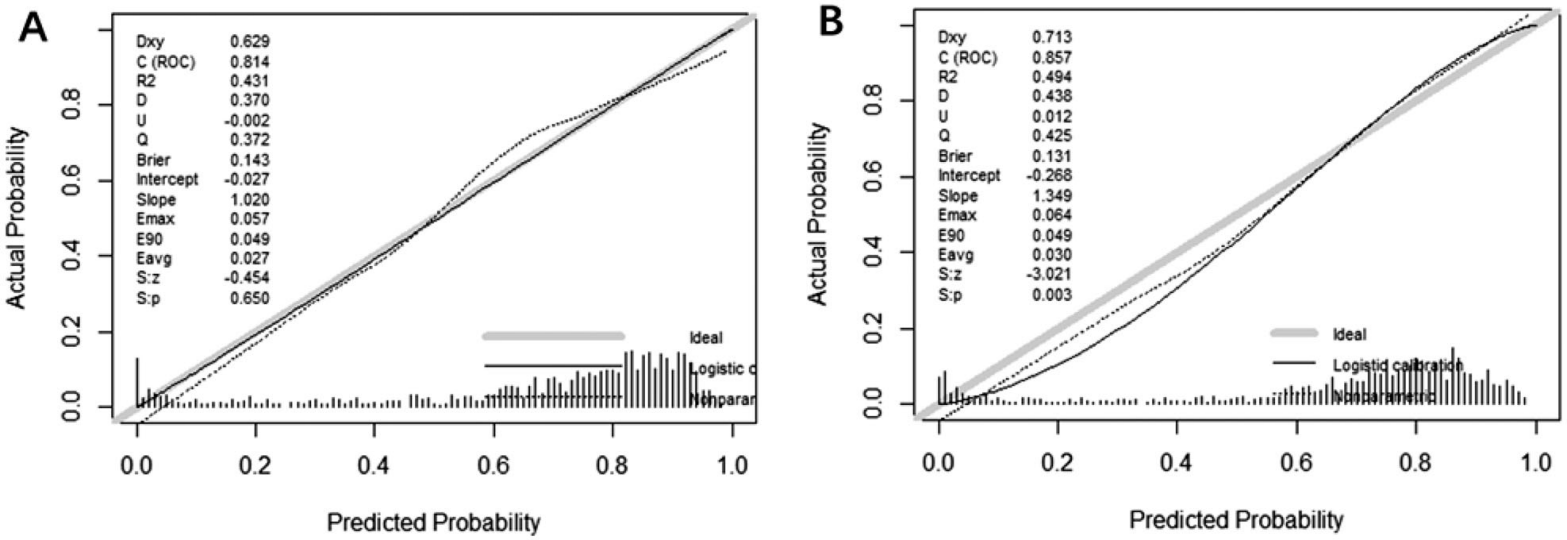
Calibration curve of the renal function recovery prediction models (A) logistic regression and (B) random forest in the training set.

A total of 846 (10%) patients were included in the model validation phase. The discrimination was appraised using an AU-ROC (Figure 3) and calibration curves (Figure 4) during the model development and validation phases. The RF model showed significantly better discrimination than the traditional logistic regression model, with a higher and more narrowed 95% confidence interval (AU-ROC, 0.8597; 95% CI 0.84–0.88 *versus* 0.8143; 95% CI 0.78–0.83) (Figure 3). Table 3 describes the model performance measures for the five models in identifying AKI recovery and non-recovery status. When considering the sensitivity and precision to predict an independent testing set, the RF model performed with a more balanced result than logistic regression.

**Table 3.** The 10-fold cross-validation model performance in the development cohort for renal function recovery.

	Logistic regression	XGBoost	Bayesian networks	Random forest	SVM
Accuracy	0.7402 ± 0.01	0.73583 ± 0.01	0.6967 ± 0.01	0.7301 ± 0.01	0.7095 ± 0.01
Sensitivity	0.6077 ± 0.02	0.5370 ± 0.02	0.4440 ± 0.03	0.5020 ± 0.03	0.4487 ± 0.02
Specificity	0.8726 ± 0.01	0.9346 ± 0.01	0.9490 ± 0.01	0.9579 ± 0.01	0.9702 ± 0.01
AU-ROC	0.8073 ± 0.01	0.8295 ± 0.01	0.7530 ± 0.01	0.8355 ± 0.02	0.8075 ± 0.01

### The model establishment and comparison of the short-term reversibility of AKI

The 8364 recovered patients were randomly split into a training and validation cohort consisting of 7619 (90%) and 846 (10%) recovered patients. Logistic regression revealed that the minimum GCS, urine volume to weight ratio within 24 h, the maximum SCr within 24 h of AKI diagnosis, the maximum BUN within 24 h, the maximum lactate within 24 h, the minimum anion gap, antibiotic duration, vancomycin, vasopressin, phenylephrine, furosemide, and ventilation within 24 h of AKI diagnosis were significantly associated with renal function recovery (Table 4).

**Table 4.** Univariate and multivariate logistic regression analyses for short-term reversibility of renal function.

	Univariate analysis		Multivariate analysis	
Variables	OR (95% CI)	*p*	OR (95% CI)	*p*
Age	1.01 (1.00–1.01)	.002	–	–
Male gender	0.92 (0.77–1.08)	.287	–	–
Ethnicity	0.96 (2.52–3.07)	.810	–	–
BMI	0.98 (0.95–1.00)	.198	–	–
Cardiovascular disease	0.91 (0.85–0.98)	.012	–	–
Chronic pulmonary disease	0.94 (0.79–1.13)	.531	–	–
Diabetes	1.07 (0.89–1.28)	.494	–	–
Sepsis	0.35 (0.28–0.43)	<.001	–	–
GCS min	1.13 (1.10–1.15)	<.001	1.08 (1.06–1.11)	<.001
SAPS II	0.97 (0.97–0.98)	<.001	–	–
SOFA > 4	0.42 (0.34–0.52)	<.001	–	–
HR mean 24 h	0.99 (0.98–0.99)	<.001	–	–
SBP min <90	0.73 (0.62–0.86)	<.001	–	–
MAP min 24 h	1.01 (1.00–1.01)	.006	–	–
T mean 24 h	0.98 (0.85–1.13)	0.77	–	–
SPO_2_ first mean	1.03 (1.01–1.04)	<.001	–	–
Urine volume 24 h	1.00 (1.00–1.01)	.008	–	–
Urine volume to weight ratio 24 h	1.64 (1.32–2.06)	<.001	1.39 (1.10–1.78)	.007
Post-AKI urine volume max 24 h	1.00 (1.00–1.01)	<.001	–	–
Post-AKI urine volume min 24 h	1.00 (1.00–1.01)	<.001	–	–
Baseline SCr	1.08 (1.06–1.10)	<.001	–	–
SCr min 12 h	0.40 (0.34–0.48)	<.001	–	–
SCr min 24 h	0.29 (0.24–0.36)	<.001	–	–
Post-AKI SCr min 24 h	0.57 (0.40–0.79)	.001	–	–
Post-AKI SCr max 24 h	0.34 (0.29–0.39)	<.001	0.54 (0.45–0.63)	<.001
Hemoglobin min 24 h	1.11 (1.07–1.15)	<.001	–	–
Leucocyte max 24 h	0.98 (0.97–0.99)	<.001	–	–
Platelet min 24 h	1.00 (1.00–1.01)	.001	–	–
Basophils min 24 h	0.97 (0.95-0.99)	.029	–	–
Monocyte max 24 h	1.00 (0.99–1.00)	.120	–	–
Albumin <3.5	0.63 (0.53–0.74)	<.001	–	–
BUN max 24 h	0.97 (0.96–0.98)	<.001	0.99 (0.98–1.00)	.007
Lactate max 24 h	0.82 (0.79–0.85)	<.001	0.92 (0.88–0.97)	.001
Anion Gap min 24 h	0.92 (0.90–0.94)	<.001	0.95 (0.92–0.98)	.002
Base excess min 24 h	1.07 (1.05–1.09)	<.001	–	–
Prothrombin time min 24 h	0.98 (0.98–0.99)	.001	0.99 (0.98–1.00)	.068
Sodium min 24 h	1.02 (1.00–1.03)	.030	–	–
Chloride min 24 h	1.01 (0.99–1.02)	.150	–	–
Bicarbonate max 24 h	1.07 (1.05–1.09)	<.001	–	–
Vancomycin	0.35 (0.28–0.42)	<.001	0.58 (0.47–0.73)	<.001
Antibiotic duration	0.37 (0.28–0.48)	<.001	–	–
Nephrotoxic drug	0.31 (0.23–0.42)	<.001	–	–
Vasopressin	0.28 (0.22–0.36)	<.001	0.68 (0.51–0.91)	.009
Phenylephrine	0.56 (0.48–0.67)	<.001	0.68 (0.51–0.91)	.009
Epinephrine	0.42(0.32–0.55)	<.001	0.81 (0.58–1.14)	.226
Furosemide	0.34 (0.27–0.42)	<.001	0.57 (0.45–0.72)	<.001
Post-AKI ventilation 24 h	1.48 (1.26–1.74)	<.001	1.44 (1.20–1.73)	<.001
AKI Stage	1.29 (1.09–1.54)	.030	–	–

AKI: acute kidney disease; BUN: blood urea nitrogen; HR: heart rate; MAP: mean arterial pressure; RRT: renal replacement therapy; SAPS II: Simplified Acute Physiology score II; SBP: systolic pressure; SCr: serum creatinine; SOFA score: Sepsis-Related Organ Failure Assessment Score; SPO_2_: peripheral oxygen saturation; T: temperature.

**Figure 5.**
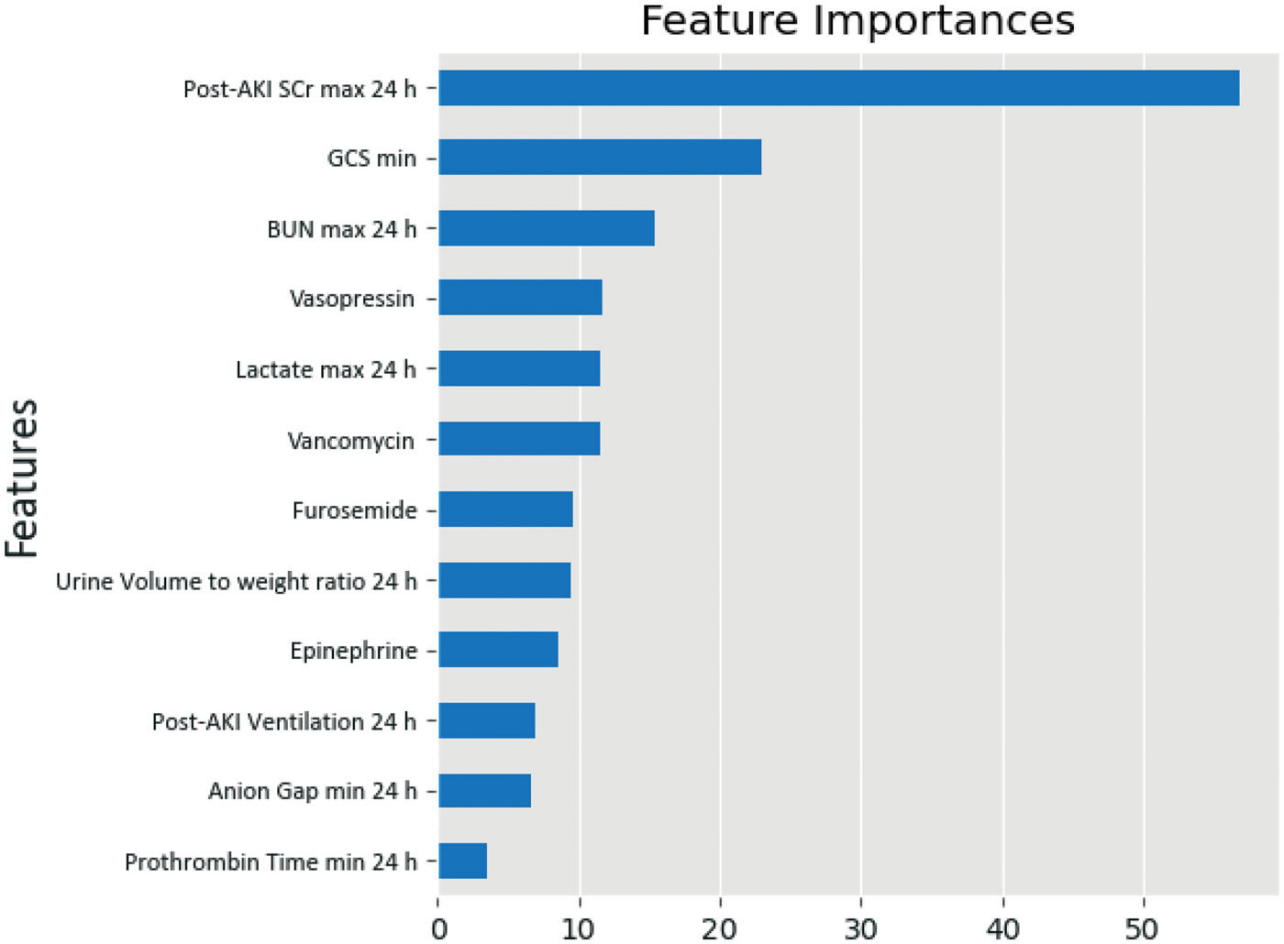
Variable importance plot for the short-term reversibility in the random forest model. GCS: Glasgow Coma Scale; SCr: serum creatinine.

We also built four machine learning models, which showed that the RF model illustrated the highest predictive performance. The maximum SCr within 24 h of AKI diagnosis, the minimum GCS, the maximum BUN within 24 h, vasopressin, the maximum lactate within 24 h, and vancomycin demonstrated notable associations with short-term renal function recovery (Figure 5). Nevertheless, for the best predictive outcomes among machine algorithms, RF was slightly better than traditional logistic regression (AU-ROC, 0.7683 ± 0.03 *versus* 0.7669 ± 0.03). Table 5 compares the models’ performances using 10-fold cross-validation.

**Table 5.** The 10-fold cross-validation model performance in the development cohort for the short-term reversibility of renal function recovery.

	Logistic regression	XGBoost	Bayesian networks	Random forest	SVM
Accuracy	0.6728 ± 0.02	0.6445 ± 0.02	0.648 ± 0.03	0.6529 ± 0.03	0.6190 ± 0.03
Sensitivity	0.4694 ± 0.04	0.3939 ± 0.04	0.4489 ± 0.05	0.3762 ± 0.06	0.2852 ± 0.05
Specificity	0.8761 ± 0.03	0.8951 ± 0.02	0.8470 ± 0.02	0.9296 ± 0.02	0.9529 ± 0.02
AU-ROC	0.7669 ± 0.03	0.7328 ± 0.03	0.7477 ± 0.03	0.7683 ± 0.03	0.7187 ± 0.03

## Discussion

The early identification of high-risk AKI populations may assist in adapting treatment in a way to avoid further renal function deterioration. Additionally, the detection of those who lack short-term reversibility may allow the determination of the optimal timing of the RRT treatment strategy for this later. By comparing conventional logistic regression with four different machine learning algorithms, we developed and tested applicable models for ICU AKI patients to help in assessing the probability of renal function recovery and predicting the short-term reversibility of AKI.

Only a few studies have predicted the prognosis of AKI with effective predictions to support the decision-making. Several clinical tools, including prediction models [19,20], urinary indices [21,22], novel biomarkers [23,24], and imaging techniques [25], have been evaluated in previous studies to predict renal recovery, namely, the progression to severe AKI.

In our model, the SCr within 24 h of AKI diagnosis might provide a significant indication for the possibility of both renal function recovery and short-term reversibility. Consistently with our findings, a recent study that enrolled 8320 critical patients with AKI pointed out the ability of the SCr for predicting persistent AKI, with the AUC of 0.74 (95% CI 0.71–0.77) [19].

The GCS score has been widely adopted as an instrument for assessing clinical severity and predicting outcomes after brain injury [26]. Moore and coworkers presented an incidence of AKI in 9% of traumatic brain injury patients with GCS scores less than 13 [27], while Zacharia et al. revealed an incidence of AKI in 23% of patients with aneurysmal subarachnoid hemorrhage [28]. In our research, those with higher GCS scores were more likely to present renal function recovery within 72 h. Therefore, it may need to highlight the indispensability of early recognition of renal risk and prompt clinicians to practice renal treatment for patients with low GCS scores. And a recent study reported that some medications can simultaneously protect the brain and kidney by inhibiting the inflammatory processes caused by brain trauma, to mitigate the incidence of AKI in neurotrauma [29].

Unsurprisingly, vancomycin showed an independent association with renal function injury [30]. It has been reported that the mechanism of vancomycin-associated AKI is the development of acute interstitial nephritis [31,32]. Our research also revealed that patients treated with vancomycin notably demonstrated poor short-term reversibility of AKI.

Positive fluid balance increased the risk for adverse outcomes and increased mortality from the vasopressin *versus* norepinephrine treatment in patients with septic shock [33], and conservative *versus* liberal fluid management strategies in acute lung injury remedies [34]. However, patients with renal function impairment were more prone to positive fluid accumulation, resulting from deterioration of kidney adjustment of water balance [35]. Bouchard et al. reported that fluid overload was associated with non-recovery of renal function in critically ill patients with AKI [36]. Similarly, in our short-term recovery model, vasopressin demonstrated harm in renal function reversibility.

Rising lactate levels revealed the insufficient perfusion, oxygen supply, and metabolism of tissues [37]. A previous study by Yan et al. pointed out that patients with poor baseline renal function have higher levels of lactate. This was consistent with our result that lactate was an indicator of kidney function, especially in predicting early AKI recovery [38].

In this study, the machine learning algorithm achieved better predictive outcomes than the conventional logistic regression, especially in predicting renal function recovery. Our study demonstrated that the SCr examination within 24 h of AKI diagnosis might provide a significant indication for the possibility of renal function recovery and recovery time. Interestingly, the GCS score not only assists clinicians in evaluating neurocognitive impairment in ICU patients but also in predicting renal function impairment duration. Furthermore, the usage of vancomycin and vasopressin were strong predictors of the short-term irreversibility of renal function.

Although this study explored the predicted model for renal function recovery with beneficial performance, it is acknowledged that there were several limitations in this study. For this large national cohort, we only validated the models with an internal dataset. In addition, novel biomarkers, which were potential predictors of renal function recovery but not routinely detected in clinical settings, were not included in our prediction models. Therefore, a multicenter prospective study should be established in the future to prove the predictive effect of the factors found in our study.

## Conclusion

In this large-cohort retrospective study, by comparing a conventional regression model with four machine learning algorithms, we developed two RF models to predict renal function recovery and short-term reversibility with high practicability and interpretability. The maximum SCr within 24 h of AKI diagnosis, the minimum GCS, vasopressin, and vancomycin were revealed to be notably associated with short-term reversibility of renal function. Consequently, predicting the recovery time of AKI may assist in assessing the likelihood of a patient needing RRT and, ultimately, could assist in determining the suitable timing for the initiation of RRT.

## Data Availability

The clinical data used to support the findings of this study were supplied by the MIMIC-III database. After completing the National Institutes of Health’s web-based course known as Protecting Human Research, participants can apply for permission to access the database.
